# Soypeptide lunasin in cytokine immunotherapy for lymphoma

**DOI:** 10.1007/s00262-013-1513-8

**Published:** 2013-12-22

**Authors:** Hua-Chen Chang, David Lewis, Chun-Yu Tung, Ling Han, Sarah M. P. Henriquez, Larry Voiles, Ivan P. Lupov, David Pelloso, Anthony L. Sinn, Karen E. Pollok, Ben O. de Lumen, Fang Li, Janice S. Blum, Shivani Srivastava, Michael J. Robertson

**Affiliations:** 1 Department of Biology, School of Science, Indiana University-Purdue University Indianapolis, 723 West Michigan Street, SL310, Indianapolis, IN 46202 USA; 2 Bone Marrow and Stem Cell Transplantation Program, Lymphoma Program, Division of Hematology/Oncology, Department of Medicine, Indiana University School of Medicine, 535 Barnhill Dr., Room 473, Indianapolis, IN 46202 USA; 3 In Vivo Therapeutics Core, HB Wells Center for Pediatric Research, Departments of Pediatrics and Pharmacology and Toxicology, Indiana University Simon Cancer Center, Indianapolis, IN USA; 4 Nutritional Sciences and Toxicology, UC Berkeley, Berkeley, CA USA; 5 Department of Mathematical Sciences, School of Science, Indiana University-Purdue University Indianapolis, Indianapolis, IN USA; 6 Department of Microbiology and Immunology, Indiana University School of Medicine, Indianapolis, IN USA

**Keywords:** Lunasin, NK, Cytokine immunotherapy, Lymphoma

## Abstract

Immunostimulatory cytokines can enhance anti-tumor immunity and are part of the therapeutic armamentarium for cancer treatment. We have previously reported that post-transplant lymphoma patients have an acquired deficiency of signal transducer and activator of transcription 4, which results in defective IFNγ production during clinical immunotherapy. With the goal of further improving cytokine-based immunotherapy, we examined the effects of a soybean peptide called lunasin that synergistically works with cytokines on natural killer (NK) cells. Peripheral blood mononuclear cells of healthy donors and post-transplant lymphoma patients were stimulated with or without lunasin in the presence of IL-12 or IL-2. NK activation was evaluated, and its tumoricidal activity was assessed using in vitro and in vivo tumor models. Chromatin immunoprecipitation assay was performed to evaluate the histone modification of gene loci that are regulated by lunasin and cytokine. Adding lunasin to IL-12- or IL-2-stimulated NK cells demonstrated synergistic effects in the induction of *IFNG* and *GZMB* involved in cytotoxicity. The combination of lunasin and cytokines (IL-12 plus IL-2) was capable of restoring IFNγ production by NK cells from post-transplant lymphoma patients. In addition, NK cells stimulated with lunasin plus cytokines displayed higher tumoricidal activity than those stimulated with cytokines alone using in vitro and in vivo tumor models. The underlying mechanism responsible for the effects of lunasin on NK cells is likely due to epigenetic modulation on target gene loci. Lunasin represents a different class of immune modulating agent that may augment the therapeutic responses mediated by cytokine-based immunotherapy.

## Introduction

Cytokine immunotherapy is one of the therapeutic strategies to harness the power of immunosurveillance to eradicate cancer cells. Numerous cytokines have been used in clinical trials to enhance anti-tumor immunity [1]. Among these cytokines, IFNγ has been recognized as its pivotal role in anti-tumor immunity by enhancing tumor immunogenicity for antigen presentation [2, 3], inducing apoptosis in tumor cells [4], promoting T helper 1 (Th1) differentiation [5, 6] and augmenting cytotoxicity of CD8+ cytotoxic T lymphocytes (CTLs) [7]. However, administration of recombinant IFNγ had disappointing outcomes in various cancer immunotherapy trials possibly due to its toxicity [8]. Efforts have focused on induction of endogenous IFNγ by natural killer (NK) or T cells following stimulation with cytokines IL-2, IL-12, IL-15, IL-18, and IL-21, which have been used individually or in different combinations [9–13]. The successful production of IFNγ is required for the efficacy of several immunotherapeutic approaches including IL-12 immunotherapy [14–16].

IL-12 can be given in biologically active doses to patients with lymphoma after high-dose chemotherapy followed by autologous peripheral blood stem cell transplantation (PBSCT) [17, 18]. However, these heavily treated patients have acquired deficiency of signal transducer and activator of transcription 4 (STAT4), which contributes to impaired production of IFNγ following IL-12 immunotherapy [19, 20]. We subsequently defined the mechanism in which acquired STAT4 deficiency in T and NK populations is caused by the chemotherapeutic regimen [21]. Failure of patient T or NK cells to adequately produce IFNγ would likely compromise the therapeutic effects during cytokine immunotherapy. The goal of this study was to develop an efficacious immune therapy that would enhance anti-tumor activity for chemotherapy-treated lymphoma patients who acquire immune dysfunctions.

In this study, we have identified a new use for the soypeptide lunasin as an immune modulating agent that synergistically works with therapeutic cytokines to enhance NK-mediated anti-tumor activity. Lunasin is a seed peptide containing 43 amino acids [22], known for its chemopreventive properties capable of suppressing tumor growth [23]. Our results have demonstrated that lunasin in combination with IL-12 or IL-2 exerts a robust synergistic effect on increasing IFNγ and granzyme B expression by NK cells; and this synergism leads to strong NK activation with enhanced cytotoxicity. Notably, the combination of lunasin and cytokines is capable of rescuing IFNγ production by NK cells from heavily treated lymphoma patients who are immune compromised. Our results suggest promise for lunasin in complementing existing modalities with IL-12 or IL-2 to improve therapeutic responses of cytokine-based cancer immunotherapy.

## Materials and methods

### Cytokines, antibodies, and lunasin peptides

Recombinant human IL-2 was obtained from Prometheus Laboratories (San Diego, CA) and recombinant human IL-12 from PeproTech (Rocky Hill, NJ). Fluorochrome-conjugated monoclonal antibodies to human CD3, CD4, CD8, CD14, CD56, FasL (CD178), IFNγ, mouse CD3, CD69, NKp46, and human/mouse granzyme B were obtained from BD Biosciences (San Jose, CA). Ficoll-Paque™ PLUS was purchased from GE Healthcare Bio-Sciences (Piscataway, NJ). The lunasin peptide with 43 amino acids was chemically synthesized with 97 % purity by LifeTein (South Plainfield, NJ), which include the following sequences: SKWQHQQDSCRKQLQGVNLTPCEKHIMEKIQGRGDDDDDDDDD. A truncated peptide (32 amino acids) lacking the RGD motif and the poly-D tail was synthesized by LifeTein. A negative control peptide with scrambled sequences (RKMELQEGI HLKKGDQNTQSQSCQPKC IQVWH) that maintains the same molecular weight to the truncated peptide was synthesized. An additional negative control peptide containing an epitope from the influenza matrix protein (M1_58–66_) which binds to human MHC class I molecules was also synthesized. All the peptides were dissolved in sterilized water at stock concentration of 5 mM.

### Human blood samples and primary cell cultures

Collection of blood was approved by the Institutional Review Board at Indiana University Medical Center, and written informed consent was obtained from each study subject. Blood samples were obtained from patients with lymphoma after treatment with high-dose chemotherapy and PBSCT. Healthy human blood samples were procured from the Indiana Blood Center (Indianapolis, IN). Peripheral blood mononuclear cells (PBMCs) were isolated using Ficoll-Paque™ PLUS, and aliquots of PBMCs were cryopreserved in liquid nitrogen. Human NK cells were isolated from normal control PBMCs using positive or negative selection kits (Miltenyi Biotech, Auburn, CA). Human B lymphoma cell line Raji cell line was obtained from American Type Culture Collection (ATCC, Manassas, VA, USA).

### Evaluation of IFNγ production

IFNγ production at the single-cell levels was evaluated using intracellular cytokine staining from PBMCs following stimulation as indicated [24]. Secreted IFNγ protein collected from the supernatant or mouse serum was measured using ELISA [19, 20].

### Analysis of gene expression

Purified human NK cells were stimulated as indicated. One day following stimulation, the cell pellets were subjected to analysis of gene expression using real-time qPCR with Taqman assay primers for *IFNG*, *CSF2*, *GZMB*, *TGFB1*, and *TGFBR2*.

### The half-maximal effective concentration (EC_50_) of lunasin

The EC_50_ of lunasin was calculated from the dose–response curve in IFNγ production using Origin Program (OriginLab, Northampton, MA). The EC_50_ is presented as mean ± SD averaged from four different normal controls.

### In vitro cytotoxicity assays

Purified human NK cells stimulated as indicated for 1 day were washed and co-cultured with target cells (Raji) at the ratio of 10:1 for 4 h at 37 °C in a 5 % CO_2_ incubator. NK-mediated lysis was analyzed using the CytoTox 96 Non-Radioactive Cytotoxicity Assay Kit (Promega, Madison, WI). For some assays, NK cells were incubated with anti-FasL-blocking antibody (NOK-1 clone, BioLegend) or the isotype control (IgG1) at 10 μg/ml for 2 h [25] followed by co-culturing with Fas-expressing target cells (Raji).

### Adoptive transfer of human NK cells in xenograft model in vivo

NOD/SCID/gc^null^ (NSG) mice (The Jackson Laboratory, Bar Harbor, Maine) at 2 months old were injected subcutaneously on day 1 with 0.5 × 10^6^ Raji cells in 0.1 ml PBS mixed with 0.1 ml Matrigel (BD Biosciences, San Jose, CA). Human NK cells isolated from healthy control donors were stimulated as indicated for 1 day. On day 2, these pre-treated NK cells were washed and injected into the tumor site (2.5 × 10^6^/mouse). Tumor growth was monitored, and the volumes were measured using standard manual calipers.

### NK activation in mice following short-term and long-term treatment

BALB/c mice received short-term (daily single IP injection for 3 consecutive days) or long-term (daily single IP injection for 5 consecutive days per week for a total of 8 weeks) treatment with PBS (−), IL-2 (1 × 10^5^ U/mouse) without (−) or with (+) lunasin (0.4 mg/kg body weight), or lunasin alone. Blood samples were collected by cardiac puncture, and serum levels of IFNγ were determined using ELISA. NK activation was analyzed from spleens in these mice using staining antibodies for surface activation marker CD69 and intracellular granzyme B. IFNγ production by NK cells was evaluated using intracellular staining from splenocytes that were incubated with GolgiPlug (Brefeldin A) for 4 h at 37 °C in a 5 % CO_2_ incubator followed by flow cytometry analysis.

### Chromatin immunoprecipitation (ChIP)

The ChIP experiment was performed using isolated human NK cells treated 1 day as indicated following the established protocol [26, 27]. Antibodies against acetyl-histone H3 (AcH3), histone H3 trimethyl Lys9 (H3K9me3), and non-immune rabbit serum were obtained from Millipore (Billerica, MA).

### Analysis of STAT4 activation by Western blot

Purified human NK cells were stimulated for 22 h. Western blot analysis was performed from total protein extracts of cultured NK cells to measure the activation of STAT4 using an anti-phospho-STAT4 (Y693) antibody (Cell Signaling Technology, Danvers, MA). The same blot was reprobed with an anti-STAT4 monoclonal antibody (BD Biosciences, San Jose, CA) for the total amount of STAT4.

### Statistical analysis

SAS/STAT (SAS Institute Inc., Cary, NC) was used to analyze the data. A mixed model was developed for analyzing the data with within-subject treatments, and the pairwise comparisons among the treatments were performed to determine the *P* values. Statistical significance between groups of mice was determined using an independent sample Student’s *t* test.

## Results

### Lunasin stimulates human NK cells to produce IFNγ

**Fig. 1:**
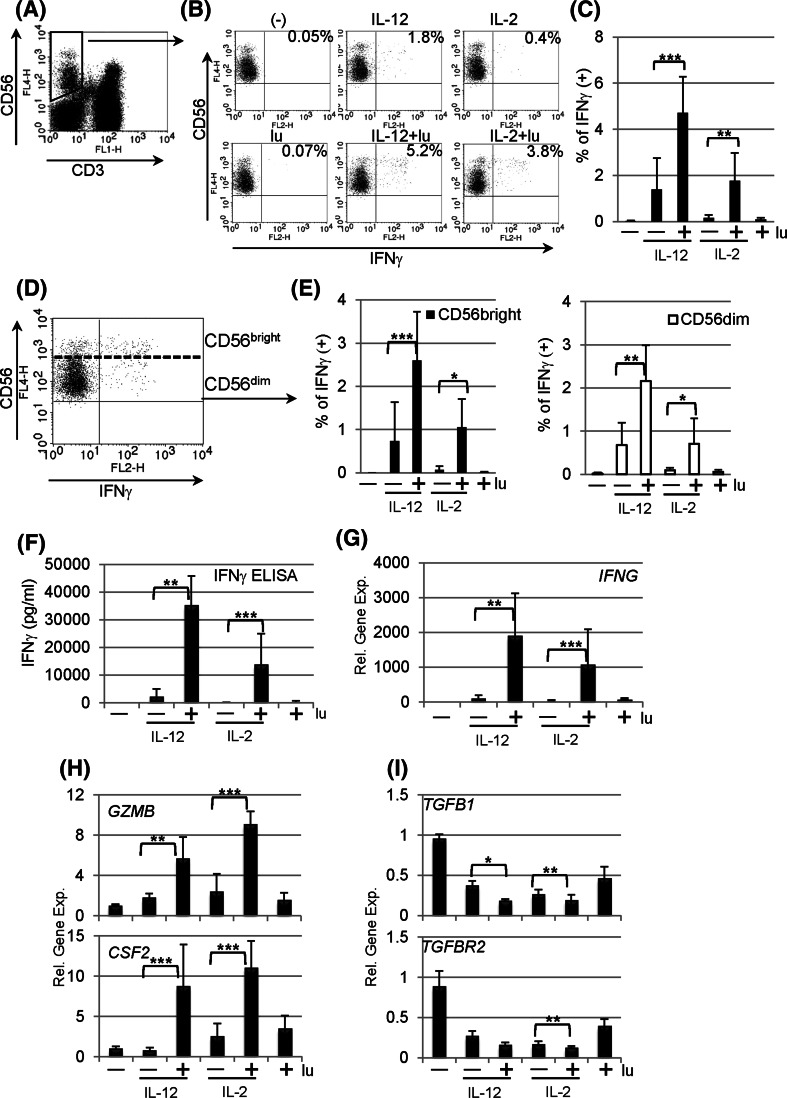
Lunasin stimulates human peripheral NK cells. Peripheral blood mononuclear cells (PBMCs) of normal controls were stimulated with medium only (−), lunasin at 20 μM (lu), cytokine IL-12 at 10 ng/ml or IL-2 at 100 U/ml, and cytokine plus lunasin for 24 h. The lunasin peptide was chemically synthesized by LifeTein (South Plainfield, NJ). The production of IFNγ at single-cell levels was analyzed using intracellular cytokine staining (**a**–**e**). At the last 6 h of stimulation, golgistop (monensin) was added to block the secretion of IFNγ. Stimulated PBMCs were surface stained with FITC-conjugated CD3 and APC-conjugated CD56 monoclonal antibodies, washed, fixed, and permeabilized. After washing, cells were incubated with PE-conjugated anti-IFNγ monoclonal antibody. Expression of IFNγ was evaluated using flow cytometry on 5,000 events of gated CD3 negative and CD56 positive NK cell populations (**a**). A representative dot plot from one donor shows the percentage of IFNγ producing NK populations following various treatments (**b**), and the averaged percentage of IFNγ producing NK populations are presented as mean ± SD from 5 different normal donors (**c**). IFNγ producing NK cells are further segregated into CD56 bright and CD56 dim populations (**d**), and the percentage of IFNγ producing CD56 bright or CD56 dim populations is averaged from the same 5 donors as in **c** and presented as mean ± SD (**e**). **f** The secretion of IFNγ by purified NK cells following stimulation was analyzed using ELISA. Freshly isolated human NK cells from PBMCs of normal controls using positive selection with CD56 magnetic beads (Miltenyi Biotec, Auburn, CA) were stimulated as in **b**. Following 1 day of stimulation, cell-free supernatants were evaluated for IFNγ production, and data are presented as mean ± SD averaged from 5 different controls. **g**–**i** Effects of lunasin on gene expression by human NK cells. The cell pellets collected from **f** were resuspended in Trizol Reagents for total RNA extraction. The first-strand cDNA was synthesized followed by real-time qPCR using Taqman assay with primers for **g** IFNγ (*IFNG*), **h** granzyme B (*GZMB*) and granulocyte–macrophage colony-stimulating factor (GM-CSF or *CSF2*), and **i** TGFβ (*TGFB1*) and TGFβ receptor (*TGFBR2*) in ABI 7300 (Applied Biosystems by Life Technologies, Carlsbad, CA). Data are presented as mean ± SD averaged from 5 different controls. *P ≤ 0.05; **P ≤ 0.01; ***P ≤ 0.001

To determine whether lunasin can induce cellular IFNγ production, PBMCs from healthy donors were stimulated with or without lunasin in the presence or absence of IL-12 or IL-2. Because IL-12 and IL-2 are known to induce the production of IFNγ by NK cells [1], these two cytokines were included in the stimulation for comparison. Following 1 day of stimulation, distinct cell populations that responded to stimulation were evaluated using intracellular staining for IFNγ. We found that CD4+ and CD8+ T populations remained negative with all stimuli (data not shown), while NK cells gated on CD3− CD56+ populations (Fig. 1a) had increased IFNγ positive cells following stimulation with lunasin and IL-12 or IL-2 compared with cytokine alone (Fig. 1b, c). CD56 bright subsets of NK cells are major IFNγ producers with regulatory functions, while CD56 dim populations exert cytolytic activity [28, 29]. We also analyzed intracellular IFNγ production by CD56 bright and dim populations (Fig. 1d), and results showed that adding lunasin to IL-12- or IL-2-cultured NK cells stimulated IFNγ production by both CD56 bright and dim populations (Fig. 1e). The effect of lunasin on NK cells was further confirmed by stimulation of purified human NK cells using either positive selection (purity ranging from 80 to 92 %) or negative selection (purity 97 %). Results showed that exposure of lunasin in combination with IL-12 or IL-2 markedly increased the levels of IFNγ secreted by purified NK cells irrespective of the method of purification (Fig. 1f). The mRNA expression of *IFNG* from the cell pellets of the same cultures correlated with the ELISA results (Fig. 1g). Consistent with intracellular staining, purified CD4+ or CD8+ T cells produced undetectable levels of IFNγ under the same stimulation conditions (data not shown). Thus, exposure of NK cells to lunasin amplifies the responsiveness of these cells to IL-12 or IL-2 as measured by IFNγ production.

### Lunasin regulates gene expression by NK cells

Because of robust synergistic effects of lunasin with IL-12 or IL-2 on inducing *IFNG* expression, we next evaluated whether lunasin was able to modulate other target genes that are regulated by IL-12 or IL-2. Results of qPCR from samples in Fig. 1g showed that adding lunasin to IL-12 or IL-2 significantly increased expression of *GZMB* (granzyme B) and *CSF2* (granulocyte–macrophage colony-stimulating factor or GM-CSF) as compared to treatment with cytokine alone (Fig. 1h). Cytokine IL-12 or IL-2 stimulation is known to downregulate *TGFB1* and *TGFBR2* expression by NK cells [30], and adding lunasin to cytokine-treated NK cultures resulted in further reduction of *TGFB1* and *TGFBR2* expression as compared to treatment with cytokines alone (Fig. 1i). Thus, it appeared that lunasin exerted synergistic effects imposed by the selected cytokine IL-12 or IL-2 on modulating expression of target genes in NK cells.

### Dose-dependent effects of lunasin in combination with cytokines

**Fig. 2:**
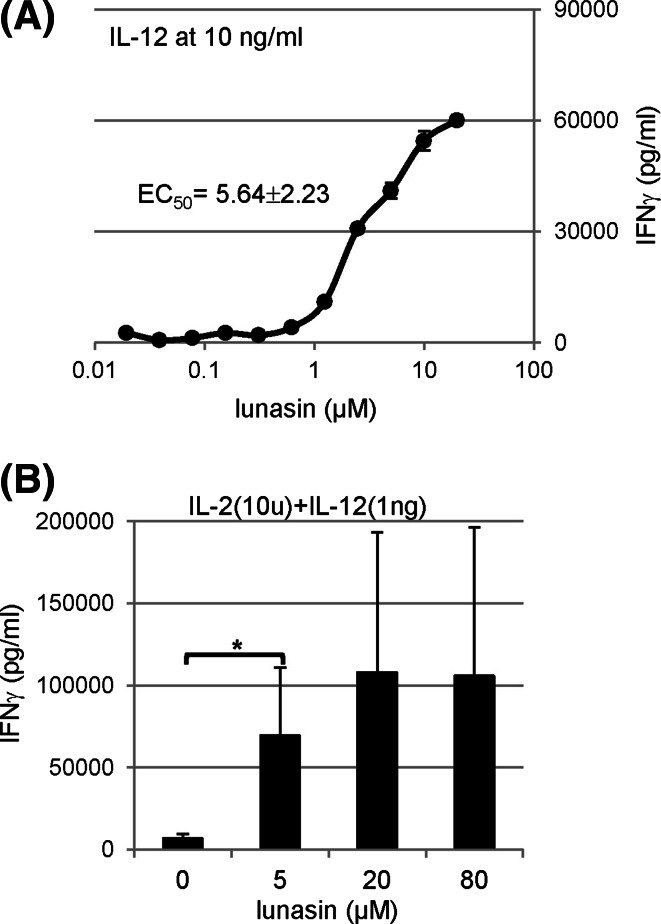
Dose-dependent effects of lunasin in combination with cytokines. Freshly isolated human NK cells from PBMCs of normal controls (described in Fig. 1f) were stimulated as indicated, and the production of IFNγ in the supernatants was determined using ELISA following one day of stimulation. **a** The half-maximal effective concentration (EC_50_) of lunasin was calculated from the dose–response curve in IFNγ production by NK cultured with IL-12 at 10 ng/ml using Origin Program (OriginLab, Northampton, MA). One representative curve based on the dose response in IFNγ production is shown. The EC_50_ is presented as mean ± SD averaged from 4 different normal controls. **b** Isolated NK cells as described in **a** were stimulated with both cytokines (IL-2 at 10 U/ml and IL-12 at 1 ng/ml) in the combination with lunasin at different concentrations ranging from 5, 20, and 80 μM. One day following stimulation, the production of IFNγ in the supernatants was determined using ELISA. Data are presented as mean ± SD averaged from 3 different normal controls. *P ≤ 0.05

To define the dose response of lunasin in combination with cytokines, we determined the EC_50_ based on the IFNγ production by IL-12-cultured human NK cells. At IL-12 concentration of 10 ng/ml, the EC_50_ for lunasin was 5.64 ± 2.23 μM (*n* = 4) (Fig. 2a). To determine whether lunasin could amplify the synergistic induction by cytokine cocktails with IL-2 and IL-12, human NK cells were cultured in both cytokines without or with lunasin. Based on the EC_50_ of lunasin, we chose its concentrations of 5, 20, and 80 μM. We found that lunasin also synergistically worked with cytokine cocktails on IFNγ production (Fig. 2b).

### Rescuing IFNγ production by NK cells from lymphoma patients post-transplant

**Fig. 3:**
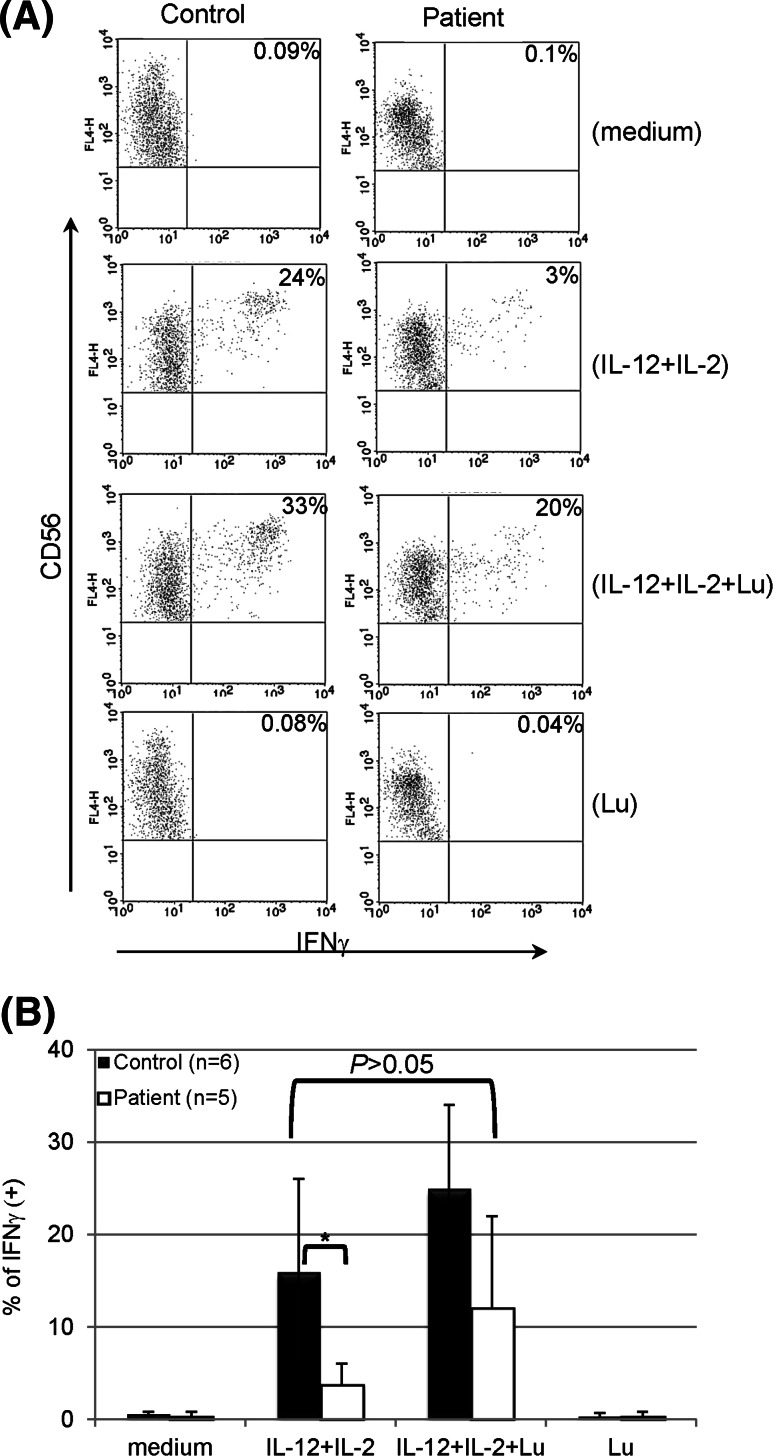
Rescuing IFNγ production by NK cells from lymphoma patients post-transplant. PBMCs of normal controls and post-transplant lymphoma patients were stimulated with medium only (−), IL-12 (10 ng/ml) and IL-2 (100 U/ml), IL-12 and IL-2 plus lunasin (lu, 20 μM), or lunasin alone for 1 day. The production of IFNγ at single-cell levels was analyzed using intracellular cytokine staining as described in Fig. 1a, and a representative dot plot from one control and patient is shown in **a**. The percentage of IFNγ positive NK populations (CD3 negative and CD56 positive) are presented as mean ± SD averaged from 6 normal controls and 5 patients (1 patient at 6–12 month and 4 patients at 3–6 months post-transplant) (**b**). *P ≤ 0.05

As reported previously, STAT4 deficiency was observed in lymphoma patients after PBSCT [21]. Stimulation of patient PBMCs with both IL-12 and IL-2 resulted in production of IFNγ by NK populations using intracellular staining (Fig. 3a), albeit at a much lower percentage as compared to cells from normal controls (Fig. 3b). However, adding lunasin to the stimulation further increased the percentage of patient NK cells that produced IFNγ, which was similar to the level from normal controls stimulated with both cytokines (*P* = 0.446) (Fig. 3b). Thus, incorporating lunasin into cytokine-based treatment may rescue the production of IFNγ by NK cells from heavily treated lymphoma patients with acquired STAT4 deficiency.

### Lunasin augments cytotoxicity by cytokine-activated NK cells

**Fig. 4:**
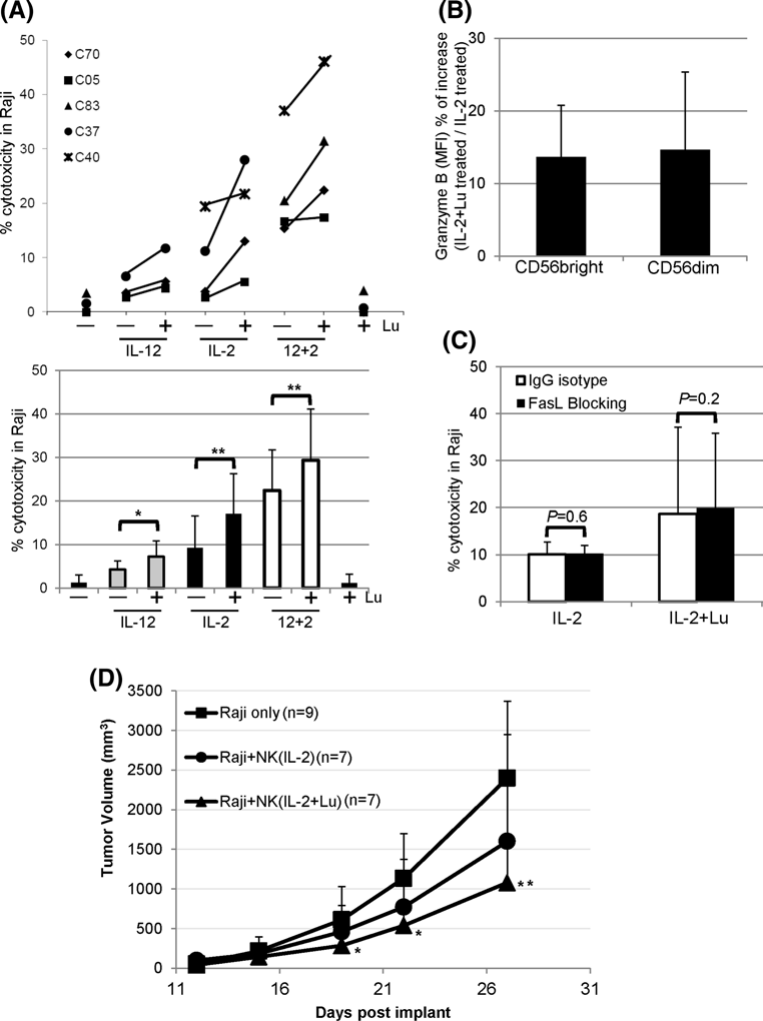
Effects of lunasin on NK cell-mediated cytotoxicity against human B lymphoma Raji cell line. **a** NK-mediated cytotoxicity in vitro. Freshly isolated human NK cells from PBMCs of normal controls using negative selection (Miltenyi Biotec) were stimulated with medium only (−), single or both cytokines (IL-12 and IL-2) without (−) or with (+) lunasin (20 μM), or lunasin alone for 1 day. Cytokines were used at suboptimum concentrations of 1 ng/ml for IL-12 and 10 U/ml for IL-2. The in vitro cytotoxicity was performed using lactate dehydrogenase (LDH)-releasing assay with the CytoTox 96 Non-Radioactive Cytotoxicity Assay Kit (Promega, Madison, WI). The effectors were co-cultured with target cells at ratio of 10:1 for 4 h at 37 °C in a 5 % CO_2_ incubator. The percentage of cytotoxicity was calculated according to the manufacturer’s instructions. Each symbol represents an individual donor, and the percentage of cytotoxicity from each donor (*n* = 5, C70, C05, C83, C37, and C40) following various treatments is depicted in the upper panel of **a**. The averaged percentage of cytotoxicity from 3 to 4 donors at each treatment is presented as mean ± SD in the lower panel of **a**. A mixed model including 6 levels of treatments was developed to analyze the data with within-subject treatments, and the pairwise comparisons among the treatments were performed to determine the *P* values. *P ≤ 0.05; **P ≤ 0.01. **b** Intracellular granzyme B staining from activated NK cells. Purified NK cells as in **a** were stimulated with suboptimum concentration of IL-2 (10 U/ml) alone or with lunasin (20 μM) for 1 day followed by intracellular staining for granzyme B. The level of granzyme B expression was analyzed from CD56 bright and CD56 dim NK populations, and the geometric mean fluorescent intensity (MFI) was obtained using flow cytometry. The MFI of bright and dim populations treated with IL-2+ lunasin was compared to IL-2, and the percentage of increase in granzyme B expression is presented as Mean ± SD averaged from 3 controls (**b**). **c** FasL-mediated killing by activated NK cells. Purified NK cells were stimulated as in **b** for 1 day followed by incubation with blocking antibody against FasL (*filled bar*) or IgG isotype control (*open bar*) for 2 h. FasL-mediated killing by activated NK cells was determined using the in vitro cytotoxicity assay as in **a**. The percentage of cytotoxicity against Raji cells is presented as mean ± SD averaged from 2 controls. **d** NK-mediated cytotoxicity in a human Raji lymphoma xenograft model. NOD/SCID/gc^null^ (NSG) mice at 2 months old were injected subcutaneously on day 1 with 0.5 × 10^6^ Raji cells in 0.1 ml PBS mixed with 0.1 ml Matrigel (BD Biosciences, San Jose, CA). NK cells were isolated from the leucopack of donors and treated with IL-2 (10 U/ml) or IL-2+ lunasin (lu, 20 uM) for 1 day. On day 2, these treated NK cells were washed and injected into the tumor site (2.5 × 10^6^ NK cells/mouse). Tumor growth was monitored, and the volumes were measured using standard manual calipers. Tumor volume (mm^3^) is presented as mean ± SD from 9 mice in the control group of Raji (no NK), and from 7 mice in the groups of Raji + NK (IL-2), and Raji + NK (IL-2+ lunasin). A mixed model with repeated measure to the data was developed using PROC MIXED in SAS program followed by pairwise comparison test of the mean differences among treatments by different days. *P ≤ 0.05; **P ≤ 0.01; relative to the control group of Raji (no NK)

NK cells cultured in medium exhibited poor cytolytic activity against Raji lymphoma cells that are resistant to NK-mediated killing (Fig. 4a). Cytokine treatment led to NK activation, which enhanced the cytotoxicity against Raji cells (Fig. 4a). Moreover, lunasin further augmented the natural cytotoxicity of NK cells that were negatively selected (97 % purity) and stimulated with suboptimum concentrations of cytokines against Raji cells (Fig. 4a).

Granzyme B is constitutively expressed by human periphery NK cells, and its level is associated with the lytic activity. IL-2 appeared to induce a higher *GZMB* by total NK cells as compared to IL-12 (Fig. 1h, upper panel). We next analyzed the effects of suboptimum IL-2 with or without lunasin on the expression of granzyme B using intracellular staining. In concert with the gene expression result, lunasin increased the protein levels of granzyme B in total NK cells cultured in IL-2 (MFI 140 ± 99 for IL-2-treated vs. 163 ± 104 for IL-2+ lunasin-treated NK; mean ± SD averaged from 3 controls). We also found that both CD56 bright and dim populations had a higher granzyme B when lunasin was included in the culture as compared to IL-2 only (Fig. 4b).

To investigate whether lunasin could affect the FasL-induced apoptosis by IL-2-cultured NK cells, we first evaluated the surface expression of FasL following 1 day stimulation. Flow cytometry analysis showed minimum effects of lunasin on surface expression of FasL in NK cells cultured in suboptimum IL-2 (3 ± 1.2 % of FasL + NK in IL-2-treated vs. 2.7 ± 0.8 % in IL-2+ lunasin-treated, *n* = 2). In addition, cultured NK cells treated with FasL-blocking antibody or the isotype control had similar killing activity against Fas-expressing Raji target cells (Fig. 4c), suggesting that lunasin had no effect on FasL-mediated killing by NK cells activated with suboptimum IL-2.

Cellular therapy using NK cells activated in vitro has been tested clinically against several tumors [31–33]. In a Raji lymphoma xenograft model, tumor growth increased over time in the control mice without transferred human NK cells (Fig. 4d). While tumor growth was attenuated at day 27 in mice receiving cytokine-activated NK cells, the group receiving NK cells activated with lunasin and cytokine had lowest tumor size when compared with the control group without transferred human NK cells (Fig. 4d).

### In vivo effect of lunasin on serum IFNγ secretion

**Fig. 5:**
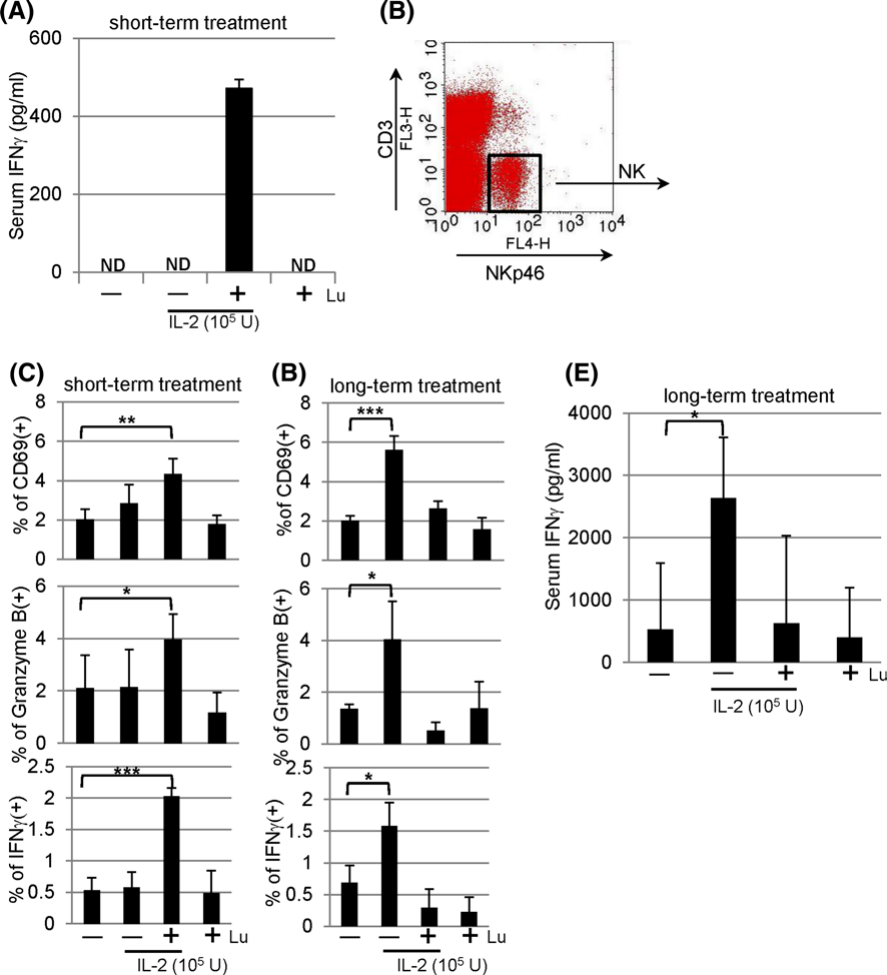
In vivo effects of lunasin. **a** Lunasin enhances the secretion of IFNγ in the serum. BALB/c mice received single intraperitoneal (IP) injection with PBS (−), IL-2 (1 × 10^5^ U/mouse) without (−) or with (+) lunasin (0.4 mg/kg body weight), or lunasin alone as indicated. Mice were killed 18 h following injection, and blood samples were collected by cardiac puncture. The serum levels of IFNγ were analyzed using ELISA. Data are presented as mean ± SD from 3 mice per group. ND, not detectable. **b**, **c** NK activation in vivo following short-term treatment. BALB/c mice received single daily IP injection for 3 consecutive days as indicated. The following day mice were killed, and spleens were collected for analysis of NK activation using flow cytometry. NK cells gated on CD3− NKp46+ populations (**b**) were analyzed for surface expression of activation marker CD69 (**c**, *upper panel*) and intracellular staining for granzyme B (**c**, *middle panel*). The production of IFNγ was analyzed from splenocytes that were incubated with GolgiPlug (Brefeldin A) for 4 h in vitro, and NK populations gated in **b** were analyzed for intracellular IFNγ expression using flow cytometry (**c**, *bottom panel*). Data are presented as mean ± SD averaged from 5 mice per group. **d**–**e** NK activation in vivo following long-term treatment. BALB/c mice received single daily IP injection for 5 consecutive days per week for a total of 8 weeks as indicated. Three days after the last injection, mice were killed, and spleens were collected for analysis of NK activation (**d**) using the same parameters as described in **c**. Blood samples were collected for analysis of serum IFNγ using ELISA (**e**). Data are presented as mean ± SD averaged from 5 mice per group. *P ≤ 0.05; **P ≤ 0.01; ***P ≤ 0.001

To assess whether lunasin could enhance IFNγ production in vivo, BALB/c mice were given single IP injection with IL-2 in the presence and absence of lunasin as indicated. Eighteen hours following the treatment, we were unable to detect serum IFNγ levels from mice receiving PBS, IL-2, or lunasin. In contrast, the combination of IL-2 and lunasin resulted in the secretion of IFNγ in the serum using lunasin at the dose at 0.4 mg/kg body weight (Fig. 5a).

### NK activation in vivo following short-term treatment

The effects of lunasin in vivo on NK activation were examined. Given the toxicity-related death caused by cytokine storm with high levels of IFNγ [34], we thus chose the regimen of IL-2 at suboptimal dose in order to keep animal alive for analysis. We analyzed NK cells gated on CD3−NKp46+ populations (Fig. 5b) from spleens of mice receiving short-term treatment (daily single IP injection for 3 days). The combination of lunasin and IL-2 resulted in significant increase in the percentage of CD69+ or granzyme B + NK cells as compared to those treated with PBS (Fig. 5C, upper and middle panels). Intracellular staining identified NK cells to be responsible for IFNγ production in mice treated with both lunasin and IL-2 (Fig. 5c, lower panel). While IL-2 at suboptimal dose had undetectable effects in this short-term treatment, adding lunasin to IL-2 was able to induce NK activation.

### NK activation in vivo following long-term treatment

Following long-term treatment (daily single IP injection for 5 days per week with a total of 8 weeks), these mice exhibited similar percentage of NK cells (CD3−NKp46+) and spleen cellularity (data not shown). Long-term treatment with IL-2 in this setting resulted in NK activation (evidenced by increased expression of CD69, granzyme B, and IFNγ; Fig. 5d) as well as increased levels of serum IFNγ (Fig. 5e). In the presence of lunasin, however, the in vivo effects of IL-2 on NK activation and serum IFNγ production were compromised (Fig. 5d, e).

### Mechanisms of synergistic effects mediated by lunasin

**Fig. 6:**
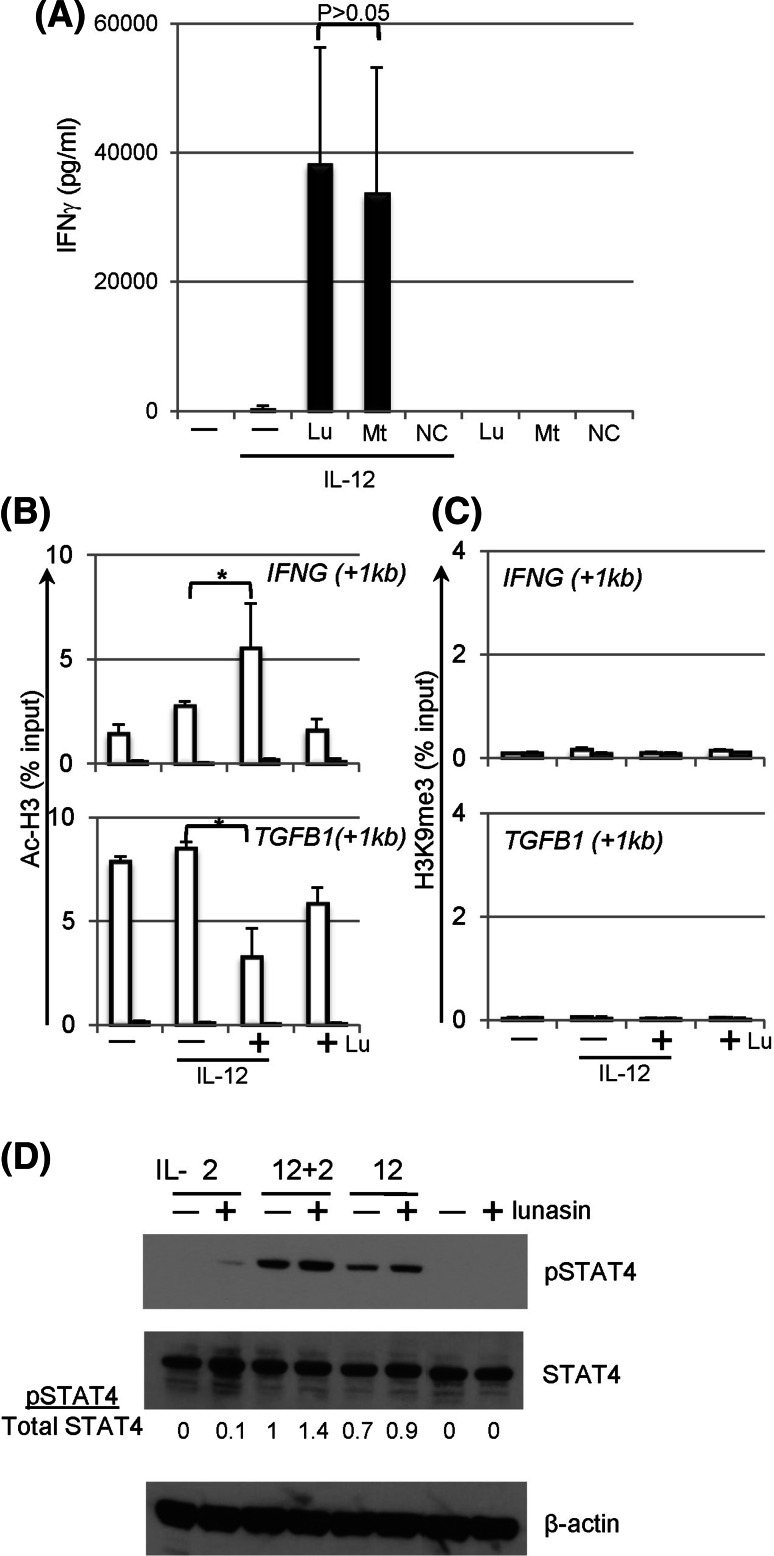
Mechanisms of synergistic effects mediated by lunasin. **a** The RGD motif and poly-D tails are not required for the synergistic effects of lunasin on IFNγ production by NK cells. Freshly isolated human NK cells (described in Fig. 1f) were stimulated with medium only (−), IL-12 (10 ng/ml) without (−) or with (+) the full-length lunasin (Lu), mutant peptide (Mt) lacking the RGD motif and poly-D tail, or negative control (NC) peptide with scrambled amino acids as well as peptides only as indicated. The concentrations of the peptides used were 20 μM. One day following stimulation, the production of IFNγ in the supernatants was determined using ELISA. Data are presented as mean ± SD averaged from 2 different normal controls. **b**, **c** Chromatin remodeling at the loci of target genes. Freshly isolated human NK cells (described in Fig. 1f) were stimulated with medium only (−), IL-12 (10 ng/ml) without (−) or with (+) lunasin (20 μM), or lunasin alone. Following 1 day of stimulation, cells were subjected to the ChIP assay. Chromatin DNA fragments were immunoprecipitated with antibodies against acetyl-histone H3 (AcH3) (**b**) and histone H3 trimethyl Lys9 (H3K9me3) (**c**) along with non-immune rabbit serum (*filled bars*) (Millipore, Billerica, MA), individually. The relative degree of histone modification of *IFNG* and *TGFB1* loci was compared by qPCR using ChIP qPCR Primer Assay for human *IFNG* (+1 kb) and *TGFB1* (+1 kb), respectively (SABioscience Qiagen, Valencia, CA). For calculation of ChIP results, the amount of immunoprecipitated DNA is normalized to the input chromatin in each reaction as a percentage of input (% input). Data are shown as mean percentage of input ± SD averaged from 3 controls. *P ≤ 0.05. **d** STAT4 activation in lunasin-cultured NK cells. Freshly isolated human NK cells from PBMCs of normal controls (described in Fig. 1f) were stimulated with single cytokine IL-2 (10 U/ml) or IL-12 (1 ng/ml), or both cytokines in the absence (−) and presence (+) of lunasin (20 μM) as well as medium and lunasin only. Activation of STAT4 was determined using Western blot of total protein extracts from cultured NK cells following 22 h of stimulation. Ratios of phospho-STAT4 to total STAT4 (pSTAT4/total STAT4) were determined from the arbitrary units of densitometry using the NIH ImageJ program as indicated. An anti-β-actin monoclonal (SC-47778) antibody (Santa Cruz Biotechnology, Santa Cruz, CA) was used for the loading control. Results shown are representative from 2 different normal controls with similar profiles

Lunasin peptide contains a RGD motif that is involved in the binding of integrins that are expressed on various cell types including NK cells [35]. To directly study the function of RGD motif in lunasin, a truncated peptide lacking the RGD motif and the poly-D tail, a negative control peptide containing scrambled amino acids, and an influenza-derived peptide that binds to MHC class I molecules, were chemically synthesized. This truncated peptide induced similar levels of IFNγ in NK cells compared with that using the full-length lunasin (Fig. 6a), suggesting the last 11 amino acids are not required for lunasin’s immune modulatory function in NK cells although they are critical for inducing apoptosis in transformed cancer cells [36]. In addition, the scrambled negative control peptide as well as influenza-derived peptide did not induce detectable levels of IFNγ (Fig. 6a, and data not shown), which ruled out the non-specific effects at the concentrations used (20 μM).

It has been shown that lunasin is capable of inhibiting the acetylation of histone H3 by p300/CBP-associated factor (PCAF), a histone acetylase enzyme [23]. Epigenetic regulation by chromatin modification is known to alter gene expression [37]. The effects of lunasin on epigenetic regulation of NK cells were examined. Consistent with gene expression profiles of *TGFB1* (Fig. 1i, upper panel), the level of acetylated histone H3 (AcH3) was negatively associated with *TGFB1* locus, and less DNA was pulled down by the anti-AcH3 antibody in NK cells treated with lunasin and IL-12 as compared to that with IL-12 alone (Fig. 6b, lower panel). Conversely, the level of AcH3 was positively associated with *IFNG* locus to a greater degree in NK cells treated with lunasin plus IL-12 than that treated with cytokine alone (Fig. 6b, upper panel). Results suggest the ability of lunasin for modulating the levels of AcH3 bound to the target genes, resulting in gene regulation.

Histone mark with tri-methylated histone H3 at lysine 9 (H3K9me3) is associated with transcriptional repression [38]. We next tested whether changes in the repressive epigenetic marker H3K9me3 were also associated with IL-12-mediated gene regulation in NK cells. Results in Fig. 6c showed a very limited binding of H3K9me3 in the gene loci including *TGFB1* and *IFNG*, suggesting that H3K9me3 was not involved in IL-12-mediated gene regulation in NK cells, and adding lunasin had no effect on levels of H3K9me3 bound to these loci.

### STAT4 activation in lunasin-cultured NK cells

STAT4 is required for IL-12-induced IFNγ production by T and NK cells [20, 39, 40], and its activation is involved in H3 hyperacetylation at *Ifng* locus [41]. In this study, we observed activation of STAT4 in human NK cells following IL-2 stimulation, albeit stronger phospho-STAT4 was induced by IL-12 (Fig. 6d). The levels of pSTAT4 were higher in the cytokine-cultured NK cells containing lunasin (Fig. 6d), suggesting that adding lunasin to cytokine-cultured NK cells enhanced STAT4 activation, which may also contribute to induction of *IFNG* by NK cells.

## Discussion

Lunasin was first described for its chemopreventive properties that have been demonstrated in cell cultures and mice models [23]. In this study, we have found that lunasin exerts synergistic effects with cytokine IL-12 or IL-2 on modulating expression of a number of genes in NK cells. This synergism results in strong NK activation with enhanced cytotoxicity, which is associated with higher levels of IFNγ and granzyme B expressed by both CD56 bright and dim populations. Adding lunasin to cytokine cocktails with both IL-12 and IL-2 was capable of rescuing the production of IFNγ by NK cells from post-transplant lymphoma patients (Fig. 3), suggesting its potential application as an alternative strategy to improve the clinical outcomes by circumventing chemotherapy-induced immune dysfunction. Taken together, our results demonstrate that the combination of lunasin and selected cytokine (designated as lunakine) is superior to cytokine alone for harnessing NK-mediated anti-tumor functions.

In addition to cytokine stimulation, NK cells can be activated by engagement of co-stimulatory molecules through direct contact with other innate immune cells [42]. Interaction between activated γδT and NK cells increases the cytotoxicity of NK cells via the CD137 engagement [43]. Thus, there is a possibility that γδT or other innate immune cells remaining in the NK cultures following negative selection (97 % purity) may respond to treatment, which in turn activate NK cytotoxicity. Future studies will need to define cell types that can respond to lunasin-based treatment, as well as target genes that are regulated by such treatment. Our results suggest that enhanced cytotoxicity of NK cells following combination treatment with lunasin and cytokine is at least in part due to up-regulation of granzyme B that involves in the granule exocytosis pathway.

Using the Raji B lymphoma xenograft model, we showed that lunakine-treated NK cells could be used in cellular therapy following adoptive transfer (Fig. 4d). However, lunasin did not increase the number of NK cells cultured in cytokines (data not shown), suggesting its limited effect on survival or expansion of NK cells in cellular therapy. Nonetheless, we verified lunasin’s in vivo effect on IFNγ production and NK activation from mice following short-term treatment with IL-2 and lunasin (Fig. 5a–c). The suppressive effect following the long-term treatment (Fig. 5d, e) was not likely caused by the reduced number of NK cells systemically as these mice exhibited similar percentage of NK cells and total spleen cellularity (data not shown). Although the 8-week treatment used in our study is not a current practice, this result raises precautions in the future use of lunasin peptide, in which immune suppression is induced following immune activation in order to maintain homeostasis and avoid the unwanted immune responses. It will be imperative to optimize the doses of each agent and duration of treatment and to evaluate the bioavailability, biodistribution, and pharmacokinetic (PK) profile for lunasin in order to fully realize its clinical potential.

The synergistic effects of lunasin with selected cytokine such as IL-12 are in part due to reducing the levels of acetyl-H3 (Fig. 6b, lower panel), which further suppresses the expression of *TGFB1* (Fig. 1i, upper panel). However, acetyl-H3 is associated with *IFNG* locus to a greater degree in NK cells treated with IL-12 plus lunasin than those treated with IL-12 only (Fig. 6b, upper panel). If the role of lunasin is solely dependent on inhibiting acetylation of histone, how can lunasin coordinately up- and down-regulate expression of different gene loci? One possibility is that lunasin can bind not only deacetylated but also acetylated histone proteins depending upon the preconditioned chromatin structure created by the selected cytokine such as IL-12. To identify histones that can physically interact with lunasin, we screened over 384 unique histone modifications using histone peptide assays (Active Motif, Carlsbad, CA). Indeed, lunasin is capable of binding to deacetylated H3 as well as H3 modifications that are associated with both gene activation and repression (data not shown). It has been shown that the level of acetyl-H3 is augmented following stimulation with IL-12, which contributes to induction of *IFNG* by NK cells [44]. We thus speculate that lunasin binds to the histone mark (acetyl-H3) created by initial cytokine exposure such as IL-12 [44], and this binding protects the target gene loci from changing its epigenetic state, and results in maintaining and stabilizing a nucleosome structure favorable for promoting transcription of target genes.

The molecular mechanisms mediated by lunasin require further investigation. Nonetheless, our results suggest that lunasin likely acts as an epigenetic modulator, which synergistically works with cytokines on regulating expression of susceptible genes in NK cells. Our discovery for the novel property of lunasin represents a different class of immune modulating agent that may augment the therapeutic responses by cytokine-based immunotherapy.
